# The Capacity of Holstein-Friesian and Simmental Cows to Correct a Negative Energy Balance in Relation to Their Performance Parameters, Course of Lactation, and Selected Milk Components

**DOI:** 10.3390/ani11061674

**Published:** 2021-06-04

**Authors:** Ilona Strączek, Krzysztof Młynek, Agata Danielewicz

**Affiliations:** Faculty of Agrobioengineering and Animal Sciences, University of Natural Sciences and Humanities in Siedlce, 08-110 Siedlce, Poland; ilonastraczek1984@gmail.com (I.S.); agata.danielewicz@uph.edu.pl (A.D.)

**Keywords:** cattle, body condition score, fatty acids, urea, β-hydroxybutyrate, metabolism, urea in milk

## Abstract

**Simple Summary:**

The aim of the study was to analyse the ability of Simmental (SIM) and Holstein-Friesian (HF) cows to correct a negative energy balance (NEB). NEB dynamics were assessed based on the content of NEFA in the blood; a reduction in body condition score; and levels of C16:0, C18:0 and C18:1 in the milk. The efficiency of liver metabolism was evaluated based on the content of BHBA in the blood and urea in the milk. The rate of changes was analysed during lactation, with assessments of daily yield, production at peak lactation and its duration, and changes in selected milk components. The results indicated that the most significant changes took place up to the peak of lactation. During this time, the values for parameters characterizing NEB were similar in both breeds. After the peak of lactation, the body condition score of SIM cows was restored more quickly. HF cows, on the other hand, achieved greater milk production and reached peak lactation earlier, but they were less capable of correcting the NEB, as indicated by the higher content of non-esterified fatty acid and β-hydroxybutyrate as well as C16:0, C18:0, and C18:1 in most cases. Their milk also contained more urea. The dynamics of NEB were found to be linked to the level of leptin, which has an anorectic effect. The results of the study indicate the great potential of Simmental cows and may facilitate the search for solutions for the more efficient exploitation of their potential.

**Abstract:**

A significant factor in improving the performance of dairy cows is their physiological ability to correct a negative energy balance (NEB). This study, using Simmental (SIM) and Holstein-Friesian (HF) cows, aimed to assess changes in NEB (non-esterified fatty acid; body condition score; and C16:0, C18:0, and C18:1) and its effect on the metabolic efficiency of the liver (β-hydroxybutyrate and urea). The effects of NEB on daily yield, production at peak lactation and its duration, and changes in selected milk components were assessed during complete lactation. Up to peak lactation, the loss of the body condition score was similar in both breeds. Subsequently, SIM cows more efficiently restored their BCS. HF cows reached peak lactation faster and with a higher milk yield, but they were less able to correct NEB. During lactation, their non-esterified fatty acid, β-hydroxybutyrate, C16:0, C18:0, C18:1, and urea levels were persistently higher, which may indicate less efficient liver function during NEB. The dynamics of NEB were linked to levels of leptin, which has anorectic effects. Its content was usually higher in HF cows and during intensive lactogenesis. An effective response to NEB may be exploited to improve the production and nutritional properties of milk. In the long term, it may extend dairy cows’ productive life and increase lifetime yield.

## 1. Introduction

During the post-partum period, dramatically increasing milk yield leads to the destabilization of the energy balance [1]. This process involves the liver, in which a sharp increase in metabolism, including of lipids and glycogen, takes place [2,3], as does the detoxification of ammonia. One of the consequences of the disturbance in homeostasis, on average up to the peak of lactation, is a temporary decrease in feed intake. This initiates changes associated with a negative energy balance (NEB) [4,5,6]. Lipolysis mainly leads to the release of palmitic (C16:0), stearic (C18:0), and oleic (C18:1) acid [7,8]. These increase the pool of non-esterified fatty acid supplied to the liver [9], in which they are oxidized and distributed in the form of low-density lipoproteins [2]. By inducing spontaneous lipolysis, NEB can adversely affect the metabolic efficiency of the liver, causing the excessive generation of β-hydroxybutyrate (BHBA) and the retention of triglycerides in the hepatocytes [10,11]. This affects the functions of the liver [12], in which the pathways of lipid metabolism, the detoxification of ammonia, and glucogenesis cross. The interactions of these pathways can impair the metabolism of these compounds [13], which may increase losses of nitrogen with the milk. This is not without significance for the ammonia detoxification pathway in the liver. An increase in ammonia levels in the blood may induce excessive lipid retention in the liver [14]. During NEB, this may result in the excessive catabolism of amino acids in the liver and increased losses of nitrogen into the environment. Studies by Knob et al. (2021) [6] and Lopreiato et al. (2019) [15] showed that the metabolic changes associated with NEB can also be influenced by the breed of cows. Therefore, the production potential of popular dairy cattle breeds remains an important area of research—especially regarding the effect of their physiological responses on production parameters and the duration of their productive life. The aim of the study was to analyse the ability of Simmental and Holstein-Friesian cows to correct a negative energy balance. We hypothesized that a less dynamic NEB at the start of lactation makes it possible to obtain more favourable production effects over a complete lactation.

## 2. Materials and Methods

The study was carried out on two breed groups (BGs) of dairy cows: 25 Holstein-Friesian (HF) cows at farm A, and 25 Simmental (SIM) cows at farm B. During the lactation period, the data were collected from the same cows. All cows in the experiment were subjected to the same number of periodic observations. The average yield (LSM ± SE) of the cows was 8390 kg ± 48 for HF and 7956 kg ± 38 for SIM, and the average lactation number (LSM ± SE) was 2.5 ± 0.23 for HF and 2.9 ± 0.43 for SIM. The cows were from two farms and constituted separate experimental groups. They were evaluated on average from the 6th day postpartum. The length of lactation (number of days) was defined as the period from calving to the day milking was discontinued, when average production had fallen to 10 kg of milk. The experiment was lasted until the completion of lactation.

### 2.1. Feeding and Housing of Cows

In both breed groups, the test cows were kept tethered. The cows had direct access to feed and water (open drinkers), and their living conditions met the requirements of good production practice.

Nutrient requirements were established based on information about feed quality—chemical analyses [16], the approximate body weight of the breed groups, and forecast milk production for the analysed stages of lactation: days 6–100 (SL I), 101–200 (SL II), and >200 (SL III). The nutritional value of the feed was determined by chemical analyses performed a few days prior to each stage of lactation. The percentages of particle sizes in the feed were determined at the same time. The body condition score (BCS) of each cow was also assessed before each stage of lactation using a 5-point scale (LSM ± SE): HF = 2.72 ± 0.42 and SIM = 2.78 ± 0.54. A loss of body condition was expressed as a percentage (LBCS%) in relation to the body condition score determined 5 days before calving. The average from three independent scores was calculated. The BCS was used as a subjective indicator of NEB. Nutrient requirements and the balancing of the diet were determined according to feeding standards for ruminants [17] and the INRAtion software, version 2.xx. HF cows were fed in a total mixed ration (TMR) system, with the ingredients mixed in a feed wagon. The SIM groups were fed in a partial mixed ration (PMR) system, in which the feed components were placed directly on the feed platform and mixed. The cows were fed three times a day (on average every 8 h) using feed pushing. The basal portion of the feed ration was calculated for cows with a body weight of 650 kg and expected milk production of 25 L. The production mix was introduced in cows exceeding this production value. The composition of the diet was similar in both herds. Table 1 shows the average content of nutrients in the daily ration of the experimental dairy cows in each stage of lactation. The ingredients of the daily cow diet are presented in Table 2.

The average amount of uneaten feed in each group was calculated based on weighing twice a month on average in each study period. On this basis, the average dry matter intake (DMI) was calculated. All diet components were included in the DMI calculation. The average percentages of particle sizes in the diet (PSPS sieves) were as follows: >19 mm (7%), 8–19 mm (52%), 4–8 mm (19%), and ≤4 mm (22%). About two weeks before calving, the cows received a preparatory diet. To rule out the effect of the farm, a cluster analysis that took the actual energy intake and DMI (considered to be the main factors inducing NEB) into account was performed. The analysis showed high similarity between the farms for these parameters during the stages of lactation: 0.782–0.921 in the case of housing and 0.789–0.829 for the diets used on the farms.

### 2.2. Sample Collection and Analyses

Milk samples of about 250 mL were collected using a calibrated milk meter (DeLaval) that simultaneously measured the amount of milk. Milk from morning and evening milking was combined into one sample. The samples were stored in refrigerated conditions (4 °C ± 0.5). Milk was collected, on average, from the 6th day postpartum until the end of lactation: in SL I, at approximately 30, 60, and 90 days of lactation (3 times); in SL II, at approximately 130 and 170 days; and in SL III, at about 230 and 270 days, for a total of 350 milk samples. The contents of protein, fat, lactose, dry matter, and urea in the milk were determined using the Bentley Combi 150 (Bentley Instruments, Inc., Chaska, MN, USA). The analysis was performed in a laboratory accredited by the Polish Accreditation Centre.

The fatty acid (FA) profile of the milk fat was determined by gas chromatography (Agilent 6890 N). Fat was extracted by the Röse-Gottlieb method [18]. The transmethylation of FA to methyl esters (FAME) was carried out at 70 °C ± 0.5 (Thermo heat block). The GLC modules were an autosampler, a split/splitless injector (split 1:5), and a flame ionization detector (FID). Separation was carried out on a 100 m, 0.250 mm column (HP-88; SN:UST458414H, Agilent Technologies Inc., Santa Clara, CA, USA). The temperature programme; injector and FID had the following run: 250 °C; furnace—95 °C (5 min); 120 °C (15 °C/min—15 min); 210 °C (25 °C/min—30 min); and 250 °C (20 °C/min—5 min). Carrier gas flow (He) was set at 5.0 mL/min. The identification of FA and determination of their percentages were based on retention times (reference Supelco 37.No:47885-U; Sigma Aldrich, St. Louis, MO, USA) in the Agilent Tech GC Chemstation A09.03 software. The following FA groups were distinguished: SCFAs—short-chain fatty acids; LCFAs—long-chain fatty acids; SFAs—saturated fatty acids; MUFAs—monounsaturated fatty acids; PUFAs—polyunsaturated fatty acids; and UFAs—unsaturated fatty acids. In addition, the contents of the (C18:0), oleic (C18:1), and palmitic (C16:0) acids in the structure of milk fat were examined. Among others, these FAs were aNEB markers.

Blood for analysis was drawn before morning feeding from the jugular vein. Due to the potential effect of stress on milk yield, blood was taken 24 h after milk was collected for analysis. Blood was collected at 30, 60, and 90 days of lactation (SL I), at 130 and 170 days (SL II), and at 230 and 270 days (SL III) for a total of 350 samples: 3 (SL) × 2 (BG) × 2 (samples) × 25 (animals). Test tubes with sodium fluoride and sodium heparin (Medlab-Products Ltd., Raszyn, Poland ) were refrigerated. Blood for glucose determination was placed in ice. Glucose content in the blood was measured using original Randox kits (Randox Laboratories Ltd., Crumlin, UK) and a UV–Vis spectrophotometer (Varian Inc., Palo Alto, CA, USA). Samples for the determination of BHBA (β-hydroxybutyrate) were centrifuged at 1500× *g* at 4 °C for 20 min. The supernatant was collected and stored at −75 °C ± 1.0 until BHBA analysis using original Randox kits (Randox Laboratories Ltd., Crumlin, UK) and a UV–Vis spectrophotometer (Varian Inc., Palo Alto, CA, USA). The plasma samples were analysed for leptin levels using a bovine-specific ELISA kit (EIAab, Wuhan, China). The share of NEFA was determined as the sum of C16:0, C18:0, and cis-9 C18:1. All samples were measured in triplicate.

### 2.3. Statistical Analysis

The statistical analysis of the results was performed with the Statistica 12.0 software. Analysis of variance was carried out in a general linear model (GLM) with repeated measures. For each BG, HF and SIM, the model included the effect of the three periods of lactation (SL): days 6–100 (SL I), 101–200 (SL II), and >200 (SL III); the interaction between the BG and SL; and the random effect of the cow. The effect of feeding technique (TMR or PMR) was verified by cluster analysis using the k-means method. The model took the actual energy intake, DMI, and effect of farm into account. The results are presented as means (LSM) and standard error (SEM). The significance of differences between means was estimated by Duncan’s test at *p* ≤ 0.05. Correlations (r) between selected parameters were estimated using the Pearson correlation model (*p* ≤ 0.05).

## 3. Results

HF cows usually had a higher daily milk production (DMP) than SIM cows (Table 3). In the stage with the highest milk production (SL I), the loss of body condition was similar in cows from both breed groups. During this period, the average LBCS was −11.80% (*p* ≤ 0.05). SIM cows, however, were able to restore their BCS faster, as from SL II, their LBCS was on average −2.1 percentage point (p.p.) smaller (*p* ≤ 0.05). Up to SL II, the DMP level remained similar. After this period, a significant decrease in DMP was noted (SL III). The average difference was 7.9 kg (*p* ≤ 0.05). As lactation progressed, a slight increase in the BCS was observed (*p* ≤ 0.05). In the first two stages of lactation, however, the restoration of body condition was slower. This was indicated by the differences in the LBCS between analysed stages of lactation: 1.2 between SL I and SL II and 7.7 p.p. between SL II and SL III (*p* ≤ 0.05).

The data in Table 4 show that over the course of lactation, there was a downward trend in the contents of NEFA and BHBA (*p* ≤ 0.05), whose levels were usually higher in HF cows. Differences between breed groups were usually greater in the first two stages of lactation than in SL III—on average, 19.65 µmol ·L^−1^ in the case of NEFA and 0.121 mmol·L^−1^ for BHBA (*p* ≤ 0.05). The changes in glucose content were also greater during these stages. Glucose content decreased up to SL II and then increased (*p* ≤ 0.05). In the SIM group, however, it decreased less (0.052 mmol·L^−1^; *p* ≤ 0.05) while the content of leptin, which exerts an anorectic effect, decreased more (0.19 ng·ml^−1^; *p* ≤ 0.05). Leptin content was also influenced by the SL. The differences between the stages of lactation indicated the greatest decrease in leptin content up to SL II, on average 0.14 ng·mL^−1^ (*p* ≤ 0.05), after which it remained at a similar level until the end of lactation. A higher urea nitrogen in milk (MUN) content, on average by 7.9 mmol·L^−1^ (*p* ≤ 0.05), was noted in the HF cows in SL I (Table 4). Generally, in subsequent stages of lactation, its level decreased (*p* ≤ 0.05), and in SL III it, was similar in both groups. Compared to that of HF cows, the milk of SIM cows had a higher DM content. However, in both BGs, there were no significant differences that indicated the influence of SL.

The milk of the SIM cows contained more SCFA (Table 5). The differences relative to HF ranged from 0.23 to 0.74 p.p. (*p* ≤ 0.05) and were smaller up to SL II. Changes in the content of SCFA during lactation showed a downward trend (*p* ≤ 0.05). The reverse trend was noted for LCFA, whose content was higher in the milk of HF cows, on average from 0.86 p.p (SL I) to 3.39 p.p (SL III). Higher contents of C16:0, C18:0, and C18:1 (treated as markers of NEB) were noted in the milk of HF cows. The greatest changes were observed in SL I and II. The differences between breed groups ranged from 0.12 p.p. in the case of C18:0 in SL III to 1.51 p.p for C16:0 in SL I (*p* ≤ 0.05). Differences between stages of lactation indicated that as lactation progressed, the share of C16:0 and C18:0 gradually declined (*p* ≤ 0.05). The levels of MUFA and PUFA, including C18:2, in the milk of the breed groups were only slightly higher in SIM cows and after the peak of lactation. In the case of these fractions, however, no clear patterns were confirmed (Table 5). The same was true for UFA. In the case of SFA, C18:1, and C18:2, gradual decreases in their contents in milk were observed over the course of lactation. The differences between stages were confirmed at *p* ≤ 0.05 for SFA and *p* ≤ 0.05 for C18:1 and C18:2. A different trend was noted in the case of the effect of lactation on PUFA and UFA. Their contents increased over the course of lactation, which was confirmed at *p* ≤ 0.05 in both cases.

The results presented in Table 6 indicate that the day of peak lactation and milk production on that day (DMP-PY) were negatively associated with the content of leptin in the blood and BSC. This was indicated by the correlation coefficients, which ranged from −0.318 to −0.452 (*p* ≤ 0.05). These parameters, however, were positively correlated with the contents of NEFA, C16:0, C18:0, and C18:1, whose release from the adipocytes was increased during NEB. These values ranged from 0.286 to 0.543 (*p* ≤ 0.05). These markers were positively correlated with BHBA (0.797–0.295; *p* ≤ 0.05) and MUN (0.711 and 0.449; *p* ≤ 0.05). The content of leptin, which exerts an anorectic effect, was positively correlated with the LBCS (0.399) and markers of NEB (0.547–0.629; *p* ≤ 0.05). A negative correlation was noted between leptin and MUN (−0.455; *p* ≤ 0.05).

## 4. Discussion

The correction of disturbances of energy homeostasis induced by intensive lactogenesis is a major challenge for the physiology of dairy cows. A lower feed intake at this time intensifies NEB and increases lipolysis [4,19]. Though a considerable portion of NEFA in cows is derived from the diet, Gross et al. (2013) [4] indicated that the intensity of daily production also has a major impact. In their opinion, the rate of change in the NEB and triglycerides, including NEFA, released from the adipocytes increases up to the peak of lactation, thus increasing the NEFA level. The consequence of this is of the inability of liver to completely use acetyl CoA from the beta oxidation of NEFA for gluconeogenesis because other intermediates of the Krebs cycle are limited for the excessive load of acetyl CoA. Finally, this situation may be conducive to the generation of excess BHBA in the liver and affects the further course of lactation [13,20]. In a study by Knob et al. (2021) [6], the BCS of Simmental cows was, on average, 1 point higher than that of Holstein cows during lactation. As in the present study, Knob et al. (2021) [6] observed the greatest loss of the BCS up to the peak of production. However, in Simmental and Simmental crossbreds, body condition relative to the BCS before calving was reduced by 12–18%, while in Holsteins, it fell by 20–24%. The stronger body condition of Simmental cows was explained by research of Yan et al. (2006) [21] and Ledinek et al. (2018) [22], in which nutrients were more evenly distributed between the lactogenesis and restoration of the BCS in cows with lower production potential. In the present study, we observed a similar relationship between NEB markers and daily yield in both breeds. The observed trends were confirmed by their significant correlations with DMP and the length of lactation. Knob et al. (2021) [6] found that the genetic group influenced yield at the peak of lactation and the time when the peak was attained. In comparison with Holstein cows, the production potential of the Simmental breed was much lower, as these authors noted a reduction in daily yield by about the fourth week of lactation. In our study, the reduction in DMP occurred much later. This difference could be explained by the much lower milk yield of the breeds analysed in our research and the study by Knob et al. (2021) [6]. Our study also showed a greater production potential in HF cows. SIM cows, on the other hand, had a greater capacity to correct the NEB, and after the peak of lactation, their BCS was more quickly restored. In this respect, the results of our study were consistent with those reported by Knob et al. (2021) [6]. Furthermore, Knob et al. (2021) [6] showed that the interaction of breed and the stage of lactation only affects NEB markers (NEFA and BHBA) to a certain extent. In contrast with our results, Knob et al. [6] found that these differences began to vanish from the fourth week of lactation. A study by Ingvartsen and Andersen (2000) [23] showed that due to genetic determinants, feed consumption in early lactation has a minor influence on milk production. Yan et al. (2006) [21], however, found that diet quality remains a significant factor by maintaining body condition during lactation and allowing cows to meet their genetic potential. According to Friggens et al. (2007) [24], changes in milk production during lactation are mainly determined by hormone metabolism, which determines the distribution of energy resources released from fat stores. In a study by Ledinek et al. (2018) [22], differences in the yield between breeds began to vanish in late lactation. Like Friggens et al. (2007) [24], they observed a decrease in hormones involved in milk secretion. Ledinek et al. (2018) [22] and Friggens et al. (2007) [24] observed a decrease in NEFA and an increase in glucose content as lactation progressed. In light of research by Ledinek et al. [22], Yan et al. (2006) [21], and Friggens et al. (2007) [24], the effects of breed and lactation on the content of the anorectic hormone leptin noted in our study are not without significance. Ledinek et al. (2018) [22] noted a higher BCS and a faster restoration of body condition to the state immediately after calving in cows of the Fleckvieh breed in comparison to HF. The differences in leptin content noted between breeds in our study and its positive correlation with the LBCS indicated that its anorectic function may delay the restoration of the BCS. According to Ledinek et al. (2018) [22] and Yan et al. (2006) [21], genetic potential is not without influence on changes in the BCS. In a study by Stengarde et al. (2008) [25], the trends in glucose content were similar to those noted in our study, and its level depended, in part, on the intensity of lactogenesis. Our study also showed that lower glucose content is accompanied by a greater loss of the BCS and a higher NEFA content. These results were in contrast to those reported by Knob et al. [6]. Though they found the highest glucose content in Simmental cows, there was no significant difference in comparison with the Holstein breed. The function of the NEB and dry matter intake were also affected by the level of glucose present in the body. Glucose content is generally lower during periods of limited feed intake and at high concentrations of NEFA [20]. Šamanc et al. (2015) [26] showed that glucose concentration was usually lower in cows with a greater loss of the BCS. In their research, the glucose content in these cows was 0.16–0.72 mmol L^−l^ lower and NEFA content was 0.12–0.20 mmol L^−1^ higher. This could be explained by the reduced glucogenic efficiency of liver cells, especially in cows with a greater reduction in the BCS and with a higher rate of BHBA production [27]. This is in agreement with research by Loor et al. (2007) [28], in which induced ketosis led to a marked decrease in glucose content and a sudden increase in NEFA. Drackley et al. (2001) [3], however, did not find glucose concentration to be dependent on NEB. In that study, the glucose levels in groups of cows differing in NEFA (as an indicator of NEB) content were similar, and no relationship was shown between the contents of glucose and insulin. The correlation coefficients obtained in our study indicated that the glucose level in the blood was negatively correlated with leptin. It was also strongly positively correlated with the BCS and strongly negatively correlated with DMP and markers of NEB. Cows with a greater NEB—especially in terms of BCS loss and increased levels of C16:0, C18:0, and C18:1—had lower glucose levels. The higher levels of these FAs may be explained by research by Stoop et al. (2009) [29], in which cows with NEB had increased contents of C16:0 and C18:0. According to the authors, this may indicate an increased mobilization of body fat reserves to provide substrates for the de novo synthesis of FA. Our study also showed that NEB can cause changes in the content of certain FA fractions, and the magnitude of these changes may be associated with both the lactation stage and breed. Research by Weber et al. (2013) [20] and Bastin et al. (2011) [30] also showed an increase in the proportion of LCFA, mainly up to the peak of lactation. After this time, they observed a stronger increase in the content of SFAs, including C18:0. In our study, we noted a slight decrease in this fraction as lactation progressed. However, we found that the stage of lactation affected the share of UFAs and that it was higher in the milk of Simmental cows than in the milk of HF. Roche et al. (2009) [31] and Ducháček et al. (2020) [19] demonstrated that the milk of cows with a mild NEB and a less severe loss of the BCS had a lower content of SFA. This may explain the results of our study, in which SFA content was lower following the period of intensive milk production and the reduction in the NEB. The differences we noted between breeds may, to some extent, be explained by research by Bastin et al. (2011) [30] that showed that changes in the structure of milk fat may be influenced by individual traits and genetic potential. The effect of the rate of production, resulting from the genetic potential of the breed, on the FA profile was also confirmed by Samková et al. (2014) [32] and Młynek et al. (2021) [33]. On the other hand, studies by Kay et al. (2005) [27] found that selection for milk yield was not the main factor determining the FA profile of milk. Petit and Côrtes (2010) [34] reported a positive correlation between SFA and an increase in the level of BHBA produced in the liver, but they noted these changes in cows fed ground flax seeds. This diet caused an increase in the share of MUFAs and PUFAs in the milk. In our study, in which both breeds received a similar diet, Simmental cows produced milk with a more favourable FA profile and also had lower BHBA and NEB markers. Furthermore, the milk of Simmental cows usually contained less urea (MUN), another product of metabolic changes in the liver [35]. However, this situation may have been influenced by the greater intake of biodegradable nitrogen, as the HF cows, due to their greater production, may have ingested more concentrate feed to compensate for their greater milk yield. MUN content is determined by numerous factors [36,37]. Studies by Kohn et al. (2002) [38] and Spek (2013) [39] indicated that a large portion of it arises from protein supplied with the diet. Following the detoxification of ammonia in the liver, it can then be eliminated, in part with the milk [40]. Of course, maintaining a balance between the intake of protein and energy in the diet plays a major role [41]. Our study, however, indicated that NEB also has a significant effect, as we observed higher MUN values in cows with faster BCS losses and higher contents of NEB markers, which were metabolized, in part, in the liver. The NEB’s function is also associated with the level of glucose present in the body. Glucose content is generally lower during periods of limited feed intake and at high concentrations of NEFA [20]. Šamanc et al. (2015) [26] showed that the glucose concentration was usually lower in cows with a greater loss of the BCS. In these cows, glucose content was 0.16–0.72 mmol L lower and the NEFA content was 0.12–0.20 mmol L higher. This can be explained by the reduced glucogenic efficiency of liver cells, especially in cows with a greater reduction in the BCS and a higher rate of BHBA production [40]. This is in agreement with research by Loor et al. (2007) [28], in which induced ketosis led to a marked decrease in glucose content and a sudden increase in NEFA. Drackley et al. (2001) [3], however, did not find glucose concentration to be dependent on NEB. In that study, the glucose level in groups of cows differing in NEFA content was similar, and no relationship was shown between the contents of glucose and insulin. In contrast with our study, Blum et al. (1983) [42] showed no differences in blood glucose levels between genetic groups, including HF and Simental. In their opinion, variation in glucose content is mainly influenced by the stage of lactation. However, a study by Djokovic et al. (2011) [43] indicated that it also depends on daily milk production. This may explain the differences obtained in our study between the HF and Simmental breeds, which had different intensities of lactogenesis in the first stage of lactation. In our study, higher glucose levels were usually noted in cows with smaller losses of the BCS and lower levels of NEFA and BHBA. At the same time, leptin content was lower in these cows, which may indicate a link with stronger appetite [5].

## 5. Conclusions

Breed influenced daily milk production up to about 100 days of lactation. After this time and until the end of lactation, the differences between NEB markers were much smaller. Cows of the SIM and HF breeds had similar dynamics of body condition loss up to the peak of lactation. In the SIM breed, however, the BCS was more quickly restored to the level noted immediately before calving. The lower urea content in the milk of SIM cows may be explained by their slightly lower production, as well as by their NEB markers. This may indicate differences arising from the potential of these breeds in terms of the physiological capacity to cope with NEB. The results indicated that the anorectic effect of leptin may play an important role in determining parameters characterizing NEB because higher leptin levels were noted during periods of intensive lactogenesis and in HF cows. The results provide a source of information that can be useful in finding solutions that could lead to the more efficient exploitation of the production potential of popular breeds of dairy cows.

## Figures and Tables

**Table 1:** Chemical components and balancing of the diet in each stage of lactation and breed group (day/cows).

Nutrient Components	Stage of Lactation (SL)/Breed Group (BG)					
	I		II		III	
	HF	SIM	HF	SIM	HF	SIM
Crude protein (%)	16.37	15.86	16.93	16.05	14.94	12.52
Dry matter (%)	42.39	41.85	44.09	42.33	38.73	35.85
Crude fibre (%)	19.17	18.52	18.46	18.41	20.60	20.42
Crude fat (%)	2.51	2.31	2.46	2.19	2.69	2.31
Crude ash (%)	8.01	7.84	8.01	7.89	8.08	7.95
Starch (%)	22.71	21.50	22.64	21.99	21.39	21.52
Acid detergent fibre—ADF (%)	22.77	21.24	22.20	21.56	24.10	22.98
Neutral detergent fibre—NDF (%)	39.49	38.74	38.57	37.44	41.54	39.42
Physically effective NDF—peNDF(%)	30.58	29.77	28.34	28.11	36.09	35.72
UFL	21.53	20.52	23.51	22.41	17.68	15.85
PDIN (g)	2459	2341	2766	2527	1854	1698
PDIE (g)	2201	2148	2459	2374	1684	1577
Energy (MJ NEL):						
Requirement	151.8	148.5	166.8	159.8	124.9	115.9
Intake	150.3	147.2	167.4	160.9	125.9	116.9
Balance	−1.5	−1.3	+0.6	+1.1	+0.2	+0.9
Dry matter intake—DMI (kg/day)	21.49	23.75	23.89	25.07	19.54	20.09

Stage of lactation: days 6–100 (SL I), 101–200 (SL II), and >200 (SL III); HF—Holstein-Friesian; SIM—Simmental; PDIN—protein digested in the small intestine, calculated from feed nitrogen (N) available in the rumen, PDIE—protein digested in the small intestine, calculated from feed energy (E) available in the rumen.

**Table 2:** Ingredients of the dairy cow diet in each stage of lactation and breed group (herd).

Nutrient Components	Stage of Lactation (SL)/Breed Group (BG)					
	I		II		III	
	HF	SIM	HF	SIM	HF	SIM
Maize silage (kg)	22.5	22.5	22.5	22.5	22.5	22.5
Haylage (kg)	12.4	12.4	12.4	12.4	12.4	12.4
Ground rapeseeds (kg)	1.45	1.5	1.03	0.9	-	-
Straw (kg)	-	-	0.2	0.2	0.5.	0.5
Hay (kg)	0.5	0.5	0.8	0.8	1.6	1.6
Production mix * (kg)	6.0	5.4	6.0	5.4	3.5	3.5

Stage of lactation: days 6–100 (SL I), 101–200 (SL II), and >200 (SL III); HF—Holstein-Friesian; SIM—Simmental; * Composition of the production mix (%): crushed maize kernels—15.5; barley—10.0; triticale—10.0; oats—16.0; Krowimix 18–2.5; ground rapeseeds—18.0; NaCl—0.3; CaCO_3_—2.3; mineral compound supplement—0.7.

**Table 3:** Production parameters and body condition of cows depending on the breed group and stage of lactation.

Parameters	Breed Group (BG)/Stage of Lactation (SL)						SEM	*p*
	HF			SIM				
	I	II	III	I	II	III		
Number of cows (n)	25	25	25	25	25	25		SL
Day of lactation (day)	40 ^c^	165 ^b^	285 ^a^	45 ^c^	162 ^b^	282 ^a^	6.4	*
DMP (kg)	30.9 ^a^	29.8 ^a^	21.5 ^c^	27.6 ^b^	27.3 ^b^	20.5 ^c^	0.4	*
BCS (points)	2.40 ^d^	2.41 ^d^	2.63 ^b^	2.45 ^d^	2.52 ^c^	2.71 ^a^	0.3	*
LBCS (%)	−11.8 ^a^	−11.4 ^a^	−3.3 ^c^	−11.9 ^a^	−9.3 ^b^	−2.5 ^c^	0.2	*

Significance of the differences in the results within the breed group (BG): ^abcd^ *p* ≤ 0.05 and stages of lactation (SL): * *p* ≤ 0.05; stage of lactation: days 6–100 (SL I), 101–200 (SL II), and >200 (SL III); HF—Holstein-Friesian; SIM—Simmental; DMP—daily milk production; BCS—body condition score; LBCS—loss of BCS relative to BCS at 5 days before calving.

**Table 4:** Parameters of energy metabolism and contents of milk components depending on the breed group and stage of lactation.

Parameters	Breed Group (BG)/Stage of Lactation (SL)						SEM	*p*
	HF			SIM				
	I	II	III	I	II	III		
Blood samples (n)	75	50	50	75	50	50		SL
NEFA (µmol L^−1^)	248.4 ^a^	211.5 ^c^	178.9 ^e^	229.5 ^b^	191.1 ^d^	182.8 ^e^	2.8	*
BHBA (mmol L^−1^)	1.021 ^a^	0.978 ^b^	0.743 ^c^	0.955 ^b^	0.776 ^c^	0.647 ^d^	0.02	*
Glucose (mmol L^−1^)	2.429 ^c^	2.360 ^d^	2.638 ^a^	2.559 ^b^	2.507 ^c^	2.632 ^a^	0.01	*
Leptin (ng ml^−1^)	2.74 ^a^	2.65 ^b^	2.50 ^c^	2.59 ^b^	2.40 ^c^	2.43 ^c^	0.02	*
Milk samples (n)	75	75	75	75	75	75		
MUN (mmol L^−1^)	181.3 ^a^	167.5 ^c^	164.3 ^c^	173.4 ^b^	164.7 ^c^	162.7 ^c^	0.71	*
Fat/Protein—F/P (%)	1.23	1.21	1.21	1.22	1.23	1.21	0.54	ns
Lactose (%)	5.01	4.78	5.19	4.97	4.85	5.12	0.32	*
Dry matter—DM (%)	12.35 ^b^	12.23 ^b^	12.52 ^b^	12.70 ^a^	12.97 ^a^	12.86 ^a^	0.08	ns

Significance of the differences in the results within the breed group (BG): ^abcde^ *p* ≤ 0.05 and stages of lactation (SL): * *p* ≤ 0.05, ns—not significant; stage of lactation (SL): days 6–100 (SL I), 101–200 (SL II), and >200 (SL III); HF—Holstein-Friesian; SIM—Simmental; BCS—body condition score; BHBA—β-hydroxybutyrate; NEFA—non-esterified fatty acid (FA); MUN—urea nitrogen in milk.

**Table 5:** Contents of the main fatty acid fractions in milk depending on the breed group and stage of lactation.

Parameters	Breed Group (BG)/Stage of Lactation (SL)						SEM	*p*
	HF			SIM				
	I	II	III	I	II	III		
Number of samples (n)	75	75	75	75	75	75		SL
SCFA (%)	9.52 ^b^	8.67 ^c^	7.75 ^e^	9.73 ^a^	8.86 ^c^	8.49 ^d^	0.05	*
LCFA (%)	59.79 ^b^	60.53 ^a^	60.77 ^a^	58.93 ^c^	58.99 ^c^	57.38 ^d^	0.16	ns
Palmitic acid C16:0 (%)	25.81 ^a^	24.90 ^b^	22.09 ^c^	24.30 ^b^	24.23 ^b^	21.86 ^c^	0.15	*
Stearic acid C18:0 (%)	11.98 ^a^	11.59 ^b^	11.38 ^c^	11.67 ^b^	11.26 ^d^	11.23 ^d^	0.44	*
SFA (%)	68.93 ^b^	69.23 ^a^	68.24 ^b^	68.29 ^b^	67.57 ^c^	65.64 ^d^	0.17	*
MUFA (%)	27.93	26.74	28.19	28.59	28.97	28.95	0.51	ns
Oleic acid C18:1 (%)	2.83 ^a^	2.37 ^b^	2.20 ^bc^	2.67 ^a^	2.16 ^c^	2.02 ^c^	0.04	*
PUFA (%)	1.57 ^c^	1.68 ^c^	1.94 ^a^	1.63 ^c^	1.79 ^b^	1.96 ^a^	0.01	*
Linoleic acid C18:2 (%)	1.68 ^a^	1.71 ^a^	1.42 ^c^	1.74 ^a^	1.57 ^b^	1.53 ^b^	0.02	*
UFA (%)	29.50 ^a^	28.42 ^b^	30.13 ^a^	30.23 ^a^	30.76 ^a^	30.93 ^a^	0.12	*

Significance of the differences in the results within the breed group (BG): ^abcde^ *p* ≤ 0.05 and stages of lactation (SL): * *p* ≤ 0.05, ns—not significant; stage of lactation (SL): days 6–100 (SL I), 101–200 (SL II), and >200 (SL III); HF—Holstein-Friesian; SIM—Simmental; LCFA—long-chain fatty acid; SCFA—short-chain FA; SFA—saturated FA; MUFA—monounsaturated FA; PUFA—polyunsaturated FA; UFA—unsaturated FA.

**Table 6:** Selected parameters of production and energy metabolism, as well as correlations between them.

Parameter	LSM ± SD	Value of Correlation Coefficient, Significant at *p* ≤ 0.05						
		DMP	BCS	Leptin	NEFA	C16:0	C18:1	C18:0
Lactation (days)	307 ± 9.2	−0.464	0.462	−0.260	−0.418	−0.462	−0.333	−0.426
Peak yield (days)	65.3 ± 8.1	0.426	−0.452	−0.358	0.543	0.385	0.429	0.337
DMP-PY (kg)	33.4 ± 4.2	0.482	−0.437	−0.318	0.342	0.428	0.382	0.286
LBCS (%)	−8.4 ± 3.4	0.758	-	0.399	0.637	0.745	0.567	0.385
NEFA (µmol L^−1^)	207.1 ± 18.3	0.805	−0.471	0.586	-	0.789	0.718	0.399
BHBA (mmol L^−1^)	0.853 ± 0.217	0.810	−0.513	0.524	0.797	0.794	0.751	0.295
Glucose (mmol L^−1^)	2.519 ± 0.251	−0.831	0.238	−0.566	−0.698	−0.793	−0.696	−0.250
MUN (mmol L^−1^)	168.9 ± 18.5	0.676	−0.530	−0.455	0.711	0.685	0.643	0.449
C16:0 (%)	23.86 ± 3.34	0.975	−0.577	0.629	0.789	-	0.816	0.353
C18:1 (%)	2.37 ± 0.16	0.831	−0.438	0.547	0.717	0.816	-	0.274

DMP—daily milk production; DMP-PY—daily milk production at peak yield; MUN—urea nitrogen in milk LBCS—loss of BCS relative to BCS at 5 days before calving; BCS—body condition score; BHBA—β-hydroxybutyrate; NEFA—non-esterified fatty acid (FA); MUN—urea nitrogen in milk.

## Data Availability

The data presented in this study are available on request from the corresponding author. The data are not publicly available due to: The research was carried out in private farms. Due to the privacy of farmers, the data will be made available in the form of a database.
